# The clinical utility of C-peptide measurement in the care of patients with diabetes

**DOI:** 10.1111/dme.12159

**Published:** 2013-06-23

**Authors:** A G Jones, A T Hattersley

**Affiliations:** 1 NIHR Exeter Clinical Research Facility, University of Exeter Medical SchoolExeter, UK; 2 Diabetes and Endocrinology, Royal Devon and Exeter NHS Foundation TrustExeter, UK

## Abstract

C-peptide is produced in equal amounts to insulin and is the best measure of endogenous insulin secretion in patients with diabetes. Measurement of insulin secretion using C-peptide can be helpful in clinical practice: differences in insulin secretion are fundamental to the different treatment requirements of Type 1 and Type 2 diabetes. This article reviews the use of C-peptide measurement in the clinical management of patients with diabetes, including the interpretation and choice of C-peptide test and its use to assist diabetes classification and choice of treatment. We provide recommendations for where C-peptide should be used, choice of test and interpretation of results. With the rising incidence of Type 2 diabetes in younger patients, the discovery of monogenic diabetes and development of new therapies aimed at preserving insulin secretion, the direct measurement of insulin secretion may be increasingly important. Advances in assays have made C-peptide measurement both more reliable and inexpensive. In addition, recent work has demonstrated that C-peptide is more stable in blood than previously suggested or can be reliably measured on a spot urine sample (urine C-peptide:creatinine ratio), facilitating measurement in routine clinical practice. The key current clinical role of C-peptide is to assist classification and management of insulin-treated patients. Utility is greatest after 3–5 years from diagnosis when persistence of substantial insulin secretion suggests Type 2 or monogenic diabetes. Absent C-peptide at any time confirms absolute insulin requirement and the appropriateness of Type 1 diabetes management strategies regardless of apparent aetiology.

## Introduction

C-peptide is produced in equal amounts to insulin and can therefore be used to assess endogenous insulin secretion, including in patients who are insulin treated. Assessment of insulin secretion is potentially helpful in clinical practice: differences in glycaemic treatment requirements between Type 1 and Type 2 diabetes mainly relate to the development of absolute insulin deficiency in the former. In addition, changes in treatment requirement with time in Type 2 diabetes also primarily relate to progressive loss of insulin secretion capacity. Despite this measurement of the underlying hormone in the clinical care of those with diabetes is infrequent.

With the rise in prevalence of Type 2 diabetes in younger patients, the discovery of monogenic subtypes of diabetes requiring specific management and the development of new therapies aimed at preserving insulin secretion the measurement of insulin secretion may be increasingly relevant in clinical practice. In addition, recent advances in assays and collection techniques have made assessment of insulin secretion using C-peptide less expensive, more reliable and widely available.

This article aims to review the current evidence on the role of the measurement of C-peptide in the management of those with diabetes. We have not addressed the use of C-peptide measurement in the assessment of hypoglycaemia aetiology and the potential therapeutic uses of C-peptide, which have been extensively reviewed elsewhere [1,2].

## Methods

A literature search of PubMed (http://www.ncbi.nlm.nih.gov/pubmed) was performed for studies published up to August 2012. Keywords used in various combinations include C-peptide, diabetes, Type 1 diabetes, Type 2 diabetes, MODY, diagnosis, classification, treatment, treatment outcome, insulin resistance, prognosis, glucagon test, mixed-meal test. Articles known to the authors or cited by others were also included.

The first radioimmunoassay for C-peptide was developed in 1970 with clinical studies following shortly after [3]. We have deliberately emphasized studies reporting diagnostic performance and more recent evidence (where available) in view of improvements in the C-peptide assay (see ‘The C-peptide assay’ below) and changes in classification and treatment of diabetes over time [4].

## C-peptide as a measure of insulin secretion

The physiology of C-peptide makes it appropriate for assessing insulin secretion. Insulin is produced in the pancreatic β-cells by enzymatic cleavage of the prohormone precursor proinsulin to produce insulin and C-peptide in equimolar amounts. C-peptide has negligible extraction by the liver and constant peripheral clearance. Its half-life is longer than insulin (20–30 vs. 3–5 min) and it therefore circulates at concentrations approximately five times higher in the systemic circulation [5,6].

C-peptide is commonly used in preference to insulin measurement when assessing β-cell function in clinical practice. In patients on insulin, C-peptide measurement must be used as exogenous insulin will be detected by insulin assays [4]. Insulin produced by the pancreas is extensively (approximately 50%) first-pass metabolized by the liver, both the extent of first-pass metabolism and peripheral clearance of insulin is variable, therefore peripheral insulin levels may not accurately reflect portal insulin secretion [7,8]. Even in non-insulin-treated patients, peripheral C-peptide levels more accurately reflect portal insulin secretion than measurement of peripheral insulin [5,9–11].

C-peptide levels must be interpreted with caution in renal failure. Approximately half of C-peptide produced is removed by the kidneys, the majority of which is degraded via peritubular uptake with approximately 5% of total C-peptide produced excreted unchanged in the urine [12,13]. Therefore, blood levels of C-peptide can be falsely elevated where there is renal impairment [14]. It has also been reported that C-peptide may be cleared to variable extents by dialysis [15]. Mechanisms to correct for renal function when measuring C-peptide have been suggested, but are currently poorly validated [16].

Although C-peptide provides a robust measure of insulin secretion in a person without renal impairment, the impact of a given level of insulin secretion will depend on an individual's insulin resistance, which can vary widely. Patients with declining insulin secretion will develop diabetes earlier when they are insulin resistant rather than insulin sensitive [17]. Therefore, an obese insulin-resistant patient may have normal or high C-peptide at the presentation even if they have autoimmune Type 1 diabetes and will go on to develop absolute insulin deficiency [18,19]. This example illustrates an important difference between the use of C-peptide and islet autoantibody testing. C-peptide gives a measure of the patient's current status (does the patient produce endogenous insulin now?) and has greater utility further from diagnosis, when rapid decline is less likely. Autoantibodies are of prognostic value (will they continue to produce endogenous insulin in the future?) and have greatest utility at diagnosis [20].

## The C-peptide assay

There have been recent improvements in the C-peptide assay. Early radioimmunoassays were time-consuming (and therefore expensive), subject to interference and often imprecise [4,21,22]. The advent of highly sensitive and specific non-isotopic assays (chemiluminescence, fluorescence, etc.) utilizing monoclonal antibodies has reduced assay costs (to approximately £10 in our laboratory) and improved detection limits and reproducibility. Cross reactivity with proinsulin is generally < 10% with modern assays and of little relevance in most clinical scenarios [as proinsulin circulates in much lower concentrations than C-peptide (pmol/l vs. nmol/l)] [4,23].

Despite these advances, some limitations remain with current assays. A large number of commercially available C-peptide assays are in use worldwide and have significant variations in comparability of results and precision [24]. Optimal standardization of C-peptide measurement between laboratories has yet to be achieved, meaning C-peptide results produced by different methods, and in some cases by different laboratories using the same methods, may not be comparable, particularly at higher C-peptide concentrations [24,25]. This means that caution is needed when interpreting C-peptide values in relation to those derived from research studies that may have used other methods, particularly where a patient's result is close to a threshold value for a clinical decision.

An additional barrier to the use of C-peptide in clinical care is a lack of available reference ranges for specific populations with diabetes. Ranges quoted by many laboratories relate to the normal population and a relevant population-based reference may be lacking, particularly where a non-fasting test is used. Data quoted in published research may relate to different assays or populations and is rarely presented as a reference range.

## Units for reporting of C-peptide values

The use of very different measurement units for reporting C-peptide values can lead to confusion in clinical care. C-peptide is commonly reported in nmol/l, pmol/l or ng/ml. All values in this article are reported as nmol/l. 1 nmol/l = 1000 pmol/l = 3 ng/ml.

## Measurement of C-peptide

### Sample handling

Traditional strict requirements for handling of blood C-peptide samples may not be necessary. Many laboratories recommend immediate transport of C-peptide samples to the laboratory on ice, with rapid centrifugation, separation and frozen storage if the sample cannot be immediately processed [4,26]. This reflects concerns about stability and limits C-peptide measurement to healthcare settings with suitably equipped on-site laboratories or, alternatively, where immediate centrifugation and freezing of samples is possible.

There is increasing evidence these sample handling requirements are not appropriate. C-peptide in whole blood collected in potassium ethylenediaminetetraacetic acid (EDTA) (rather than the more commonly used serum gel) and measured using modern immunoassay analysers is stable at room temperature for at least 24 h; in contrast, C-peptide in blood collected into serum gel or plain sample tubes is stable for 6 h but shows marked degradation by 24 h [27–29]**.**

### Measurement of C-peptide in blood

When assessing insulin production, C-peptide can be measured in a fasting or non-fasting (‘random’) sample or in a formal stimulation test (e.g. after intravenous glucagon or a standardized mixed-meal test). While formal stimulation tests are most accurate and reproducible for research purposes, a fasting or non-fasting (‘random’) sample is usually suitable in clinical practice if the sampling conditions (timing relative to food and associated glucose) are known.

**FIGURE 1:**
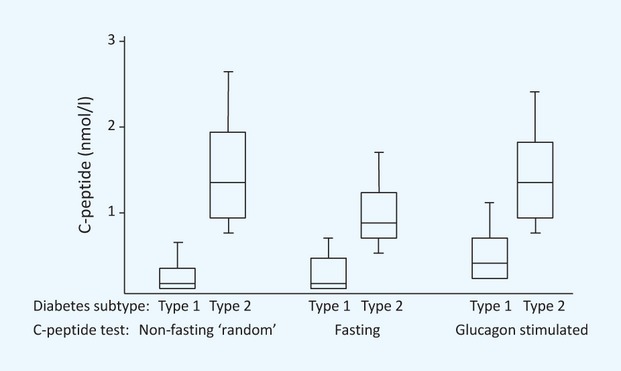
Boxplot of random non-fasting (with glucose > 8 mmol/l), fasting and glucagon-stimulated C-peptide in well-defined (on clinical features) Type 1 (*n* = 371) and Type 2 (*n* = 732) diabetes. Redrawn with permission from Berger *et al*. ([39]). Horizontal line represents median, box interquartile range, ‘whiskers’ represent 10–90% of values.

Median and interquartile ranges of C-peptide measured fasting, non-fasting (random) and after-glucagon stimulation in those meeting strict criteria for Type 1 and Type 2 diabetes in a predominantly Caucasian Swedish adult population are shown in Fig. 1. Approximate equivalent values of fasting and stimulated C-peptide for key clinical thresholds are shown in Table 1. Where C-peptide is referred to as ‘stimulated’ or ‘post-stimulation’ in these tables and throughout the article, we are referring to the absolute post-stimulation C-peptide value, rather than the increment above baseline sometimes reported, which we do not advocate for clinical use.

**Table 1:** Suggested C-peptide thresholds to support clinical decisions in patients with insulin-treated diabetes

Clinical role	Stimulated (non-fasting ‘random’/post-glucagon/mixed-meal test) (nmol/l)	Fasting (nmol/l)	Post-meal home meal urine C-peptide:creatinine ratio (nmol/mmol)
Absolute insulin deficiency/absolute insulin requirement [76]	< 0.2	< 0.08	< 0.2
Likely Type 1 diabetes/inability to achieve glycaemic control with non-insulin therapies [39,40,95]	< 0.6	< 0.25	< 0.6
Suggests Type 2 or monogenic (MODY) diabetes in a patient with presumed Type 1 diabetes > 3–5 years post-diagnosis [65,71]	> 0.2	> 0.08	> 0.2
Consider MODY/Type 2 diabetes in young onset diabetes at diagnosis [67]	> 1	> 0.4	> 1.1

Equivalent thresholds for stimulated and fasting C-peptide and urine C-peptide:creatinine ratio have been calculated from a data set of 120 research participants with insulin-treated diabetes and 90-min post-mixed-meal and fasting C-peptide and home urine C-peptide:creatinine ratio measurements using linear regression with zero origin [37,62,63]. These thresholds are approximate; values close to thresholds should be treated with great caution and may not assist clinical decision making.

Fasting C-peptide measurement is logical when assessing insulin resistance in patients not treated with insulin (see separate section). However, β-cell stimulation in the fasting state may be reduced by the hypoglycaemic effect of concomitant insulin administration [30,31]. Therefore, where assessing β-cell function, measurement of C-peptide after stimulation may be advantageous [32,33]. Correlations between fasting C-peptide and post-stimulation C-peptide are high in insulin-treated patients (*r* = 0.84–0.99) [34–37]; however, the use of stimulated C-peptide (including non-fasting ‘random’ samples) does appear to offer modestly better clinical utility [38–41].

It has been proposed that C-peptide results are corrected for concurrent glucose measurement. While this appears to better correlate with β-cell mass and glucose intolerance after islet cell transplant, there are limited published data using this approach in clinical practice, making interpretation of this ratio difficult [16,30,42]. A pragmatic approach is to measure concurrent glucose to exclude hypoglycaemia (which will suppress insulin and C-peptide) with a glucose > 8 mmol/l considered a stimulated value [31,39]. The homeostasis model assessment (HOMA) B calculation using fasting insulin and C-peptide is not advised for use in clinical practice and is not valid in those on insulin therapy [42–44].

Non-fasting ‘random’ C-peptide is likely to be the most easily performed blood test of insulin secretion in the clinical setting. A large study of C-peptide in the classification of adult diabetes suggested non-fasting random C-peptide with a concurrent glucose over 8 mmol/l was superior to both fasting and glucagon-stimulated C-peptide measurement in correctly classifying clinically well-defined Type 1 and Type 2 diabetes [39]. Random non-fasting C-peptide appears superior to fasting C-peptide in classifying diabetes based on autoantibody status [38]. However, the utility of random C-peptide measurement has otherwise been little examined. Despite this, it is likely that in many clinical situations a random (and therefore presumably imprecise) measure of insulin secretion may suffice. The spread of C-peptide levels is wide and a high or very low level will exclude or confirm severe insulin deficiency; a fasted or stimulated test could then be conducted should the result be indeterminate.

Where formal post-stimulation C-peptide measurement is desired, there is a wide range of published stimulation methods that have been used. The best evidenced are the glucagon test (serum C-peptide measured 6 min after intravenous glucagon 1 mg intravenously given in the fasting state) and mixed-meal tolerance test [serum C-peptide measured 90–120 min (or area under curve over 120 min) after a liquid mixed meal (commonly Sustacal™; Mead Johnson & Company, Evansville, IN, USA or Boost™; Nestlé Health Science, Lutry, Switzerland 6 ml/kg up to a maximum 360 ml) given in the fasting state] [34,45–49]. A definitive comparison in early Type 1 diabetes shows that C-peptide at 90 min in the mixed-meal tolerance test is more reproducible than post-glucagon C-peptide measurement and better tolerated [34]. The liquid mixed meals used in major research trials (e.g. Sustacal™ and Boost™) are not easily obtainable in many European countries, including the UK, although it is likely that preparations with broadly similar nutritional content will be interchangeable.

### Measurement of C-peptide in urine

Urine C-peptide measurement is a potentially attractive non-invasive measure of β-cell function. C-peptide is excreted in the urine through glomerular filtration and uptake from peritubular capillaries. The total quantity of C-peptide excreted in the urine per day represents approximately 5% of pancreatic secretion, compared with only 0.1% of secreted insulin [50]. The concentration in urine is typically 10–20 times higher than in plasma and the absence of proteases found in blood mean that C-peptide is more stable—at room temperature a sample collected in boric acid (standard midstream urine container) is stable for at least 72 h and a sample without preservative 24 h [51].

While many studies have demonstrated strong correlation between total 24-h urine C-peptide and serum C-peptide [33,52–55], others have shown only modest correlation [35,56–58]. There appears to be inter/intra-individual variation in the fraction of secreted C-peptide appearing in the urine [58] and urinary C-peptide clearance appears to be higher in diabetes, likely through hyperglycaemia increasing the glomerular filtration rate [56,59–61]. These concerns and the practical difficulties of 24-h urine collection have limited the use of 24-h urine C-peptide in clinical practice.

Correcting for creatinine adjusts urine C-peptide concentration for variation in urine concentration and enables the use of ‘spot’ urine samples in place of 24-h urine collection. Recent work by the Exeter group has shown 2-h urine C-peptide:creatinine ratio is highly correlated with serum C-peptide measurements in the mixed-meal tolerance test in insulin-treated diabetes (*r* = 0.82 [62] to *r* = 0.97 [63]) and with meal stimulated C-peptide in non-insulin-treated diabetes [64].

**FIGURE 2:**
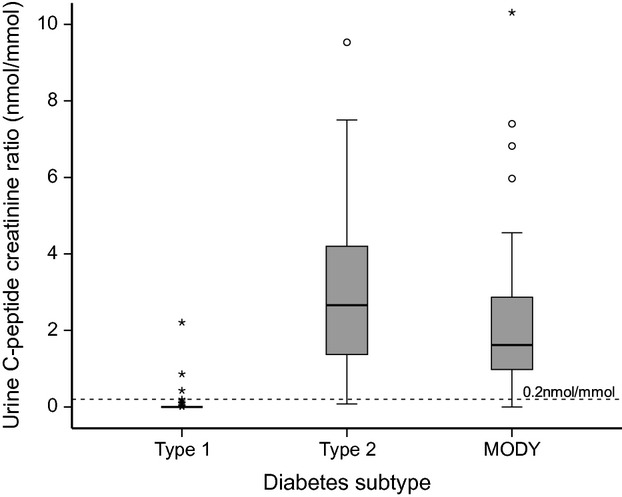
Boxplot using post-home meal urine C-peptide:creatinine ratio to discriminate Type 1 diabetes of over 5 years’ duration (*n* = 70) from Type 2 diabetes (*n* = 64) and HNF1A/4A MODY (*n* = 81). Adapted with permission from Besser *et al*. ([65]) (redrawn from original data). Cut-off of 0.2 nmol/mmol 96% sensitive and 98% specific in differentiating Type 1 diabetes from Type 2 diabetes or MODY (area under the receiver operating characteristic curve 0.99). Horizontal line represents median, box interquartile range, ‘whiskers’ represent the spread of remaining values. (o) outliers over 1.5 times the interquatile range, (*) outliers over 3 times the interquartile range.

Home samples collected in boric acid after a patients largest meal of the day and returned for analysis by post remain well correlated with mixed-meal test serum C-peptide (*r* = 0.83, combined data from reference [63] and [62], insulin-treated diabetes) and are a sensitive and specific test for the presence of significant endogenous insulin secretion and differentiating long-standing Type 1 diabetes from other diabetes subtypes (Fig. 2) [62,63,65]. Values of urine C-peptide:creatinine ratio for key clinical thresholds are shown in Table 1, alongside equivalent values of fasting and stimulated blood C-peptide. Urine C-peptide:creatinine ratio levels are 1.5-fold higher in women than men, as a result of higher creatinine levels in men; however, we do not currently advocate correction for clinical use [66].

### Summary

In summary, a range of tests are available to clinicians to assess insulin secretion. It is likely that, in the majority of clinical scenarios, a less intensive test such as non-fasting ‘random’ blood C-peptide, fasting blood C-peptide or post-meal urine C-peptide:creatinine ratio will be sufficient. If maximum accuracy is required, a mixed-meal tolerance test is best tolerated and has highest reproducibility, but is more time-consuming than a one-off sample or glucagon test.

## Clinical utility of C-peptide measurement

### Classification of diabetes

#### Differentiating Type 1 and Type 2 diabetes

An important clinical role of C-peptide is differentiating between Type 1 and Type 2 diabetes. Utility is greatest in long-standing diabetes as there may be a substantial overlap of C-peptide levels between Type 1 and Type 2 diabetes at the time of diagnosis.

Key studies reporting diagnostic performance in differentiation of Type 1 and Type 2 diabetes are summarized in Table 2. A major limitation in interpreting these studies is the lack of a gold standard for the diagnosis of Type 1 or Type 2 diabetes and, in many cases, the potential incorporation of the C-peptide result into a ‘clinical’ classification, which may lead to positive bias. Where diabetes is classified purely on the basis of the presence or absence of autoantibodies, C-peptide remains a relatively good predictor with better performance than either age of diagnosis or BMI [18,38].

**Table 2:** Summary of studies reporting diagnostic performance of C-peptide in differentiating Type 1 and Type 2 diabetes since 1990

Reference	Number, population and study design	C-peptide test*	C-peptide threshold and predictive value of values below or above threshold for diabetes subtype or islet autoantibody status	Notes
At diagnosis of diabetes				
Ludvigsson, 2012 [67]	2734 children newly diagnosed with diabetes (Type 1 95%, Type 2 or MODY 3%). C-peptide alone compared with final diagnosis incorporating clinical features and knowledge of autoantibody status, C-peptide, human leukocyte antigen (HLA) status and (in some cases) MODY genetics	Non-fasting ‘random’	< 0.2 nmol/l > 99.8% predictive value Type 1 diabetes ≥ 1.0 nmol/l 46% predictive value Type 2 diabetes or MODY	C-peptide at diagnosis a much stronger predictor of Type 2 diabetes or MODY than age or glycaemia
Thunander, 2012 [18]	1178 adults diagnosed over 20 years (mean age 66). C-peptide at diagnosis compared with presence or absence of islet autoantibodies (GAD or ICA, 4.9% antibody positive)	Fasting	< 0.6 nmol/l 30.1% predictive value autoantibodies, > 0.6 nmol/l 97.4% predictive value absence of autoantibodies	C-peptide superior to age and BMI in discriminating autoimmune and non-autoimmune diabetes
Katz, 2007 [129]	175 children with new-onset diabetes. Type 2 diabetes (15%) if obese, relative with Type 2 diabetes, ability to wean from insulin, GAD antibody negative	Fasting	< 0.28 nmol/l 98% predictive value Type 1 diabetes > 0.28 nmol/l 48% predictive value Type 2 diabetes	
Torn, 2001 [38]	486 newly diagnosed aged 15–34 years, C-peptide measured in either fasting or non-fasting ‘random’ and compared with presence of islet autoantibodies (ICA, GAD, IA-2A, 74% antibody positive)	Fasting and non-fasting ‘random’	Fasting < 0.3 nmol/l 85% predictive value autoantibodies Non-fasting < 0.3 nmol/l 94% predictive value autoantibodies Fasting > 1.0 nmol/l 75% predictive value absense of autoantibodies Non-fasting > 1.0 nmol/l 83% predictive value absense of autoantibodies	
Long-standing diabetes				
Besser, 2011† [65]	Urine C-peptide:creatinine ratio measured post-home meal in 70 patients with Type 1 diabetes (diagnosis age < 30 years, insulin from diagnosis) and 69 patients with Type 2 diabetes (diagnosis ≥ 30 years, no insulin in first post-diagnosis year)	Urine C-peptide:creatinine ratio	< 0.2 nmol/mol 98.5% predictive value Type 1 diabetes > 0.2 nmol/l 95.3% predictive value Type 2 diabetes	Long duration diabetes (Type 1 diabetes median 34 years) may account for high performance of the low threshold in predicting Type 2 diabetes
Berger, 2000 [39]	Retrospective analysis of 1093 patients with well-defined diabetes type (34% Type 1) who had had C-peptide measured in clinical care (duration at C-peptide testing not reported). Type 2 diabetes: clinicians diagnosis and no insulin for 3 years. Type 1 diabetes: clinicians diagnosis and continuous insulin for > 3 years from diagnosis	Fasting Non-fasting C-peptide with glucose > 8 mmol/l Glucagon stimulated	Fasting < 0.42 nmol/l 81.0% predictive value Type 1 diabetes Fasting > 0.42 nmol/l 91.3% predictive value Type 2 diabetes Non-fasting < 0.5 nmol/l 91.5% predictive value Type 1 diabetes Non-fasting > 0.5 nmol/l 95.3% predictive value Type 2 diabetes Glucagon-stimulated < 0.6 nmol/l 93.9% predictive value Type 1 diabetes Glucagon-stimulated > 0.6 nmol/l 77.1%	C-peptide may have influenced diagnosis. Included patients whose C-peptide was measured at or close to diagnosis
Service, 1997 [130]	346 patients with diabetes (mostly long-standing) classified as insulin-dependent diabetes (24%) and non-insulin-dependent diabetes (76%) by clinical algorithm. Clinical classification compared with classification by C-peptide—fasting < 0.17 nmol/l and increment < 0.07 indicating insulin-dependent diabetes, all other responses defined as Type 2 diabetes	Fasting and increment in mixed-meal tolerance test	Fasting C-peptide < 0.17 nmol and mixed-meal tolerance test increment < 0.07 predictive value Type 1 diabetes 77%. All other C-peptide responses predictive value Type 2 diabetes 93%	Follow-up for up to 8 years showed C-peptide classification remained stable
Prior, 1993 [41]	373 (Type 2 diabetes 114) adults with known retinopathy meeting study definitions of Type 1 diabetes (*n* = 259, diagnosis < 30 years, insulin within 1 year, weight < 120% desirable) or Type 2 diabetes (*n* = 114, diagnosis > 30 years and not on insulin or diagnosis > 40 years and weight 120% desirable)	Fasting and 90 min in mixed-meal tolerance test	Mixed-meal tolerance test C-peptide < 0.08 nmol/l = 100% predictive value Type 1 diabetes. Mixed-meal tolerance test C-peptide > 0.08 nmol/l 91% predictive value Type 2 diabetes. Fasting C-peptide < or > 0.08 nmol/l 97.4% agreement with mixed-meal tolerance test classification	Long duration of diabetes (retinopathy required for inclusion) may account for the low threshold chosen

Where not reported, predictive values have been calculated from published data.

All studies have predominantly Caucasian populations.

* Blood unless urine C-peptide:creatinine ratio stated.

† Type 1 diabetes vs. Type 2 diabetes diagnostic performance not reported—calculated from original study data.

**Table 3:** Summary of studies reporting diagnostic performance of C-peptide in identifying patients able to discontinue insulin treatment since 1990

Reference and year	Number and patient characteristics	Test type	Summary of methods	C-peptide threshold	Criteria defining successful insulin withdrawal	Predictive value of C-peptide above threshold for insulin withdrawal	Predictive value of C-peptide value below threshold for failed insulin withdrawal	Notes
Iwao, 2012 [87]	69 Japanese patients with antibody-negative Type 2 diabetes [mean HbA_1c_ 57 mmol/mol (7.4%) receiving complex insulin therapy > 1 year	Fasting, post-meal, 24-h urine	Consecutive patients invited, insulin stopped and liraglutide started in hospital. Duration 12 weeks	60 min post-meal 0.97 nmol/l	Blood glucose pre-and post-meals lower than on insulin therapy for three consecutive days and < 17 mmol/l	95%	93%	82% of ‘successful insulin withdrawal’ had HbA_1c_ 53 mmol/mol (< 7%) at 12 weeks. Some participants received additional oral therapy
Hohberg, 2009 [131]	98 adults with well-controlled [HbA_1c_ < 58 mmol/mol (7.5%)] insulin-treated Type 2 diabetes and glucagon-stimulated C-peptide ≥ 0.6 nmol/l	Glucagon stimulated	Insulin stopped and pioglitazone ± sulphonylurea commenced. No control group (C-peptide < 0.6 nmol/l not included). Duration 6 months	≥ 0.6 nmol/l for study inclusion	Not more than 5 mmol/mol (0.5%) HbA_1c_ deterioration over 6 months	77%	Not available	Small improvement (1 mmol/mol; 0.1%) in HbA_1c_ seen in the 77% who remained off insulin at 6 months
Maldonaado, 2003 [95]	103 predominantly African and Hispanic American adults presenting with diabetic ketoacidosis	Fasting and post-glucagon	Insulin reduction then cessation where American Diabetes Association glucose targets met following clinical protocol	Fasting > 0.33 nmol/l or post-glucagon > 0.6 nmol/l	Blood glucose readings < 6.7 mmol/l fasting and < 7.8 pre-bed	50%	100%	Management in specialist clinic following protocol. 3% vs, 34% recurrence diabetic ketoacidosis high vs. low C-peptide
Lee, 1999 [40]	64 adults with Type 2 diabetes, diagnosis > 35 years, BMI > 28 kg/m^2^, mean HbA_1c_ 68 mmol/mol (8.4%). Excluded if renal/liver failure, ketoacidosis	Fasting and 2 h post-100 g oral glucose	Attempted insulin withdrawal/weaning in all using metformin and troglitazone. Duration 8–12 weeks	Fasting 0.3 nmol/l, post-glucose 0.68 nmol/l	Fasting glucose < 7.8 mmol/l, pre-meal < 10 mmol/l, HbA_1c_ 64 mmol/mol (< 8%)	Fasting 90% Post-glucose 100%	Fasting 79% Post-glucose 94%	Age, BMI, duration of diabetes and HbA_1c_ not predictive of response
Bell, 1998 [132]	130 C-peptide-positive patients with diabetes duration 10–30 years on insulin < 10 years. Mean HbA_1c_ 86 mmol/mol (10%)	Fasting or non-fasting ‘random’	Metformin then sulphonylurea added, insulin gradually withdrawn. Mean duration of follow-up 6 months	> 0.27 nmol/l for study inclusion	HbA_1c_ < 86 nmol/l (10%)	77% successfully stopped insulin initially. 60% at mean 6-month follow-up	Not available	No relationship between baseline C-peptide and response seen

An additional limitation is that the development of absolute insulin deficiency is a key feature of Type 1 diabetes and more relevant marker of subtype (and treatment requirements) than clinical characteristics such as age of diagnosis and BMI, both of which increasingly overlap between Type 1 and Type 2 diabetes as obesity rates increase. For example, if age and BMI suggest Type 2 diabetes, but the patient has absolute insulin deficiency, following guidelines for Type 1 diabetes therapy (such as multiple daily injections or continuous subcutaneous insulin infusion with carbohydrate counting) are likely to be appropriate regardless of the apparent aetiology—see below ‘Detecting absolute insulin deficiency’.

**FIGURE 3:**
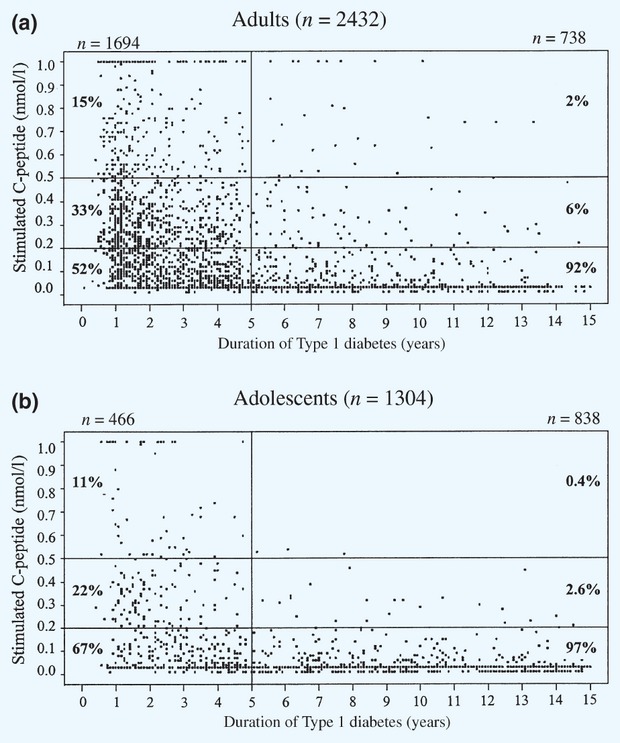
Two-hour mixed-meal test C-peptide values in relation to diabetes duration at entry screening for the Diabetes Control and Complications Trial in (a) adults aged > 18 years and (b) adolescents aged < 18 years. Reproduced with permission from Palmer *et al*. ([23]).

The variation in optimal cut-offs and predictive value between studies may reflect population differences (particularly time from diagnosis of diabetes and prevalence of Type 1/Type 2 diabetes, predictive value will depend on pretest probability) and variations in both the stimulation test and C-peptide assays used. There is substantial overlap between C-peptide levels in Type 1 and Type 2 diabetes close to diagnosis and this will be greatest in obese or older patients in whom the clinical differentiation of Type 1 and Type 2 diabetes is most difficult [18,19,67]. In Type 1 diabetes, insulin/C-peptide levels rapidly fall, therefore the utility of C-peptide testing increases from 3 to 5 years post-diagnosis, where the vast majority of patients with Type 1 diabetes will have low C-peptide levels (Figs 2 and 3) [23,65].

C-peptide levels taken within the first few years of diagnosis may be useful in confirming Type 1 diabetes if results are low (e.g. non-fasting blood C-peptide < 0.2 nmol/l with hyperglycaemia confirms severe insulin deficiency, < 0.6 nmol/l Type 1 diabetes likely). However, higher results should be interpreted with caution (particularly in the obese or those with features of insulin resistance—see ‘C-peptide as a measure of insulin secretion’) and may simply reflect continued insulin secretion seen in the early Type 1 diabetes ‘honeymoon period’. In this situation, repeated measures may be helpful.

#### Identifying patients with maturity-onset diabetes of the young (MODY)

Persistence of C-peptide is an important clinical feature of MODY. It is particularly important to identify these patients as they are commonly misdiagnosed as Type 1 diabetes and treated with insulin. More than 60% of MODY is caused by mutations in the genes *HNF1A* and *HNF4A*; these patients are very sensitive to sulphonylurea treatment and are commonly able to stop insulin treatment with improved glycaemic control [68]. Other patients with glucokinase mutations do not require glucose-lowering treatment [69]. In contrast to Type 1 diabetes, substantial insulin secretion persists in these forms of diabetes outside of the honeymoon period and the persistence of C-peptide in a patient thought to have Type 1 diabetes may be suggestive of MODY [70]. A home post-meal urine C-peptide:creatinine ratio ≥ 0.2 nmol/mmol > 5 years post-diagnosis has 97% sensitivity and 96% specificity for differentiating HNF1A/4A MODY from Type 1 diabetes (Fig. 2) [65]. A random blood C-peptide of ≥ 0.2 nmol/l in those with diabetes diagnosed under 30 years of age and > 3 years’ duration has been suggested as a criteria for consideration of MODY testing [71]. C-peptide testing is unlikely to be useful in differentiating MODY from Type 2 diabetes [65].

There are limited data available on the utility of C-peptide testing in identifying other forms of monogenic diabetes. Patients with mitochondrial diabetes may develop severe insulin deficiency [72,73] and those with monogenic neonatal diabetes commonly have absolute insulin deficiency in the absence of sulphonylurea therapy [74]. Patients with monogenic forms of diabetes associated with severe insulin resistance are likely to have raised C-peptide.

#### Summary

In summary, where there is uncertainty as to diabetes subtype, C-peptide measurement may aid diagnosis and therefore appropriate management. This is particularly relevant in long-standing (> 5 years) insulin-treated diabetes where retained substantial C-peptide secretion may be strongly indicative that Type 1 diabetes is unlikely and therefore Type 2 diabetes or MODY should be considered.

### Detecting absolute insulin deficiency

Regardless of the aetiology/classification of a person's diabetes, the awareness that a person has absolute insulin deficiency (commonly defined as < 0.2 nmol/l after a mixed-meal test or < 0.08 nmol/l fasting [37,75,76]) is important to clinical management. A person with absolute insulin deficiency will have an absolute requirement for insulin to prevent ketoacidosis and greater glycaemic instability, hypoglycaemia risk and microvascular complications [75–77]. It is logical that (regardless of whether their diabetes is autoimmune in origin) these patients may particularly benefit from ‘Type 1’ type treatments such as basal bolus insulin, carbohydrate counting or insulin pumps, should be managed as Type 1 diabetes during illness or surgery and will have reduced response to therapies acting through stimulation of endogenous insulin secretion such as sulphonylureas or incretin-based therapies.

### Treatment response

#### Treatment change in insulin-treated patients

C-peptide may help identify insulin-treated patients with sufficient β-cell function to safely replace insulin with other hypoglycaemia therapies. Early work established that stimulated C-peptide of approximately 0.3–0.8 nmol/l could differentiate insulin-requiring from non-insulin-requiring diabetes, using definitions of acceptable control very different from today, often without oral hypoglycaemic medications [77–83]. Using a lower cut-off will give greater specificity for insulin requirement, all patients with a stimulated C-peptide < 0.2 nmol/l are likely to have an absolute requirement for insulin. Studies since 1990 formally assessing insulin withdrawal (and reporting diagnostic performance) are summarized in Table 3. Cut-offs in these and earlier studies (C-peptide approximately 0.6 nmol/l stimulated and 0.3 nmol/l fasting) are unsurprisingly similar to those distinguishing Type 1 and Type 2 diabetes. It should be noted these studies have a number of limitations, including that they are generally of short duration and often use definitions of acceptable glycaemia that are far less stringent than those in use today. Few recent studies have included a group with low C-peptide, instead excluding these patients based on data from earlier research. Participants had a clinical diagnosis of Type 2 diabetes and were usually insulin treated from diagnosis or had previously been treated with only a single oral therapy.

The clinical role of C-peptide testing in this context is likely mainly to exclude absolute insulin deficiency prior to attempted insulin withdrawal in patients insulin treated from diagnosis and thought unlikely to have Type 1 diabetes or who have had long-standing insulin treatment for presumed Type 2 diabetes.

There may be an additional role to exclude severe insulin deficiency prior to addition of oral or glucagon-like peptide 1 (GLP-1) agonist therapy to insulin, particularly where there is doubt about the underlying diabetes subtype. Neither sulphonylureas nor incretin-based therapies are currently recommended for Type 1 diabetes and treatments acting wholly or partly through enhancing β-cell insulin secretion would appear likely to have less response in those who do not secrete endogenous insulin. However, direct evidence is limited. Fasting C-peptide does not appear to predict the effects of sulphonylurea withdrawal within those with Type 2 diabetes who have progressed through oral therapy to requiring insulin, this may reflect a low prevalence of absolute insulin deficiency in this population [84–86]. A high predictive utility of blood C-peptide for liraglutide response in insulin-treated patients was reported in one small study [87]; however, another has found only a small difference in C-peptide in those able and unable to replace insulin with exenatide [88].

There may be a potential future role of C-peptide testing in assisting choice of insulin regimen. Many insulin-treated patients with Type 2 diabetes achieve good glycaemic control with intermediate or long-acting insulin alone, but fast-acting mealtime insulin may be required as diabetes progresses. C-peptide is inversely associated with glycaemic variability and post-meal glucose rise in both Type 1 and Type 2 diabetes [89–92] and is inversely associated with response to prandial insulin in experimental conditions in a mixed population with diabetes [93].

#### Insulin dependence in ketosis-prone diabetes

C-peptide measurement may help to detect adult patients presenting with diabetic ketoacidosis who do not have classical Type 1 diabetes and may not require long-term insulin treatment. Patients with negative islet autoantibodies and preserved C-peptide (fasting > 0.33 nmol/l or glucagon response > 0.5 nmol/l) on resolution of ketoacidosis are likely to retain endogenous β-cell function at 1 year and in many cases achieve glycaemic control without insulin (approximately 50% in a predominantly non-Caucasian population [94]), in contrast to those with low initial C-peptide levels [95,96].

#### Treatment change in non-insulin-treated patients

There is limited evidence to support the use of C-peptide to predict treatment response in non-insulin-treated patients.

In a population with newly diagnosed Type 1 and Type 2 diabetes, very low C-peptide appears to be predictive for insulin requirement. In 244 consecutively recruited patients, low fasting C-peptide had similar predictive values for subsequent insulin treatment to positive islet cell antibodies (ICA): 80% of those with fasting C-peptide < 0.25 nmol/l required insulin over a median 31 months’ follow-up [97].

In Type 2 diabetes a double-blind trial of metformin and glibenclamide has demonstrated that achieving good glycaemic control with these agents in those with marked hyperglycaemia can be predicted by a combination of baseline glycaemia and stimulated C-peptide levels [98]. Logistic regression suggested the probability of a patient with a fasting glucose of 16 mmol/l achieving glycaemic control would vary from 15 to 85% in those with low and high C-peptide. Retrospective observational studies [99,100] using postprandial C-peptide:glucose ratio to predict future insulin treatment in Type 2 diabetes are consistent with this; however, reported test performance appears similar to that of BMI and fasting glucose [99], and clinicians knowledge of C-peptide status may have led to positive bias.

The large overlap between C-peptide levels in patients with Type 2 diabetes who do and do not require insulin for glycaemic control goes against the use of C-peptide in this context [101]. Inexpensive and effective oral hypoglycaemic therapies are available and a trial of treatment in most cases will be the most effective way of determining response. It is also not clear that those with low C-peptide would have benefited from earlier insulin therapy: low C-peptide may be associated with poor control regardless of therapy [102,103], although it has been reported that those with Type 2 diabetes and fasting C-peptide < 0.2 nmol/l have better control on insulin rather than oral treatment [104].

Evidence for a clinical role of C-peptide in predicting response to specific hypoglycaemic agents is weak. There is evidence that more insulin-resistant patients with higher C-peptide values have increased response to thiazolidinediones [105–108]. This does not appear to be the case for metformin, sulphonylureas and dipeptidyl peptidase-4 (DPP-4) inhibitors [84,105,109–111].

In summary, there is currently insufficient evidence for more than a very limited role of C-peptide in this context. There may be a role for assessment of C-peptide in assisting the decision between oral and insulin therapy in those presenting with marked hyperglycaemia, and in providing supporting evidence for prescribing pioglitazone in a patient suspected to have marked insulin resistance.

#### Glycaemic response to bariatric surgery in Type 2 diabetes

Preoperative C-peptide assessment has been shown to be associated with remission of Type 2 diabetes after bariatric surgery in an Asian population [112]. Remission rates were 55% in patients with preoperative fasting C-peptide < 1 nmol/l vs. 90% in those with fasting C-peptide > 2 nmol/l. These differences were less pronounced in the subgroup undergoing bypass (rather than restrictive) surgery.

#### Summary

The current clinical role of C-peptide in predicting treatment response is principally to exclude severe endogenous insulin deficiency in insulin-treated patients when considering insulin withdrawal or when considering the addition of therapies dependant on endogenous insulin for their action. There may be a limited role at the diagnosis of Type 2 (or undetermined subtype) diabetes with marked hyperglycaemia where C-peptide testing may support a clinical decision on initial insulin therapy.

### Determining prognosis

In Type 1 diabetes even very modest residual β-cell function as measured by C-peptide is associated with improved glycaemic control, less hypoglycaemia and substantial reductions in microvascular complications [76,113,114]. In the Diabetes Control and Complications Trial study, participants with post-mixed-meal tolerance test C-peptide levels of > 0.2 nmol/l had a 10 mmol/mol (0.9%) lower HbA_1c_ at baseline screening and markedly less incidence of retinopathy, nephropathy and hypoglycaemia [76]. In Type 2 diabetes, high C-peptide levels may be associated with features of the metabolic syndrome and increased macrovascular complications [115–117]. The relationship between C-peptide and microvascular complications in Type 2 diabetes is unclear, with an association found by some authors [115,117–120], but not others [116,121,122].

### Partial remission phase/honeymoon period in Type 1 diabetes

The preservation of insulin secretion often seen for an initial period after diagnosis of Type 1 diabetes is associated with reduced hypoglycaemia and glycaemic variability, improved HbA_1c_ and lower insulin requirements [92,123]. There may be a role for using C-peptide to monitor insulin secretion during this period in some circumstances; for example, to help explain whether a deterioration in glycaemic control relates to a decline in insulin secretion or to unrelated patient factors (such as medication adherence). A future clinical role of C-peptide testing would in pre-screening and monitoring of response for interventions to preserve endogenous insulin secretion should these come into clinical practice [23,124].

### Islet transplantation

C-peptide can be used to assist patient selection for islet cell transplantation and post-transplant monitoring [125]. C-peptide < 0.1 nmol/l (fasting and/or mixed-meal tolerance test) has been used as a criterion for islet cell transplantation and to define complete graft failure [126].

### Insulin resistance

Although fasting C-peptide can be used to derive an estimate of insulin resistance using HOMA modelling [44], and high uncorrected fasting C-peptide in the presence of hyperglycaemia may be suggestive of insulin resistance, methods based on direct insulin measurement (rather than C-peptide) are generally used for research purposes [127] and evidence for use in this clinical context is limited [128].

## Recommendations

We recommend C-peptide measurement in diabetes clinical practice predominantly in insulin-treated patients where there is uncertainty about the underlying diagnosis or consideration of a therapy requiring residual β-cell function for its mechanism of action. In this increasingly common clinical context, C-peptide may assist appropriate treatment and classification.

Numerous stimulation methods have been proposed in the literature. In most clinical practice a fasting blood C-peptide, non-fasting blood C-peptide in the presence of a glucose > 8 mmol/l or post-home meal urinary C-peptide:creatinine ratio are appropriate. Values close to clinical thresholds could be repeated or a more rigorous stimulated test (mixed-meal or glucagon tests) performed.

In a person with insulin-treated diabetes, a stimulated blood C-peptide of < 0.6 nmol/l (fasting < 0.25 nmol/l and or post-meal urinary C-peptide:creatinine ratio < 0.6 nmol/mmol) are suggestive of marked insulin deficiency and Type 1 diabetes. Values over this are consistent with short-term insulin independence in an individual who has not previously ‘failed’ non-insulin therapy, but may occur in the Type 1 diabetes honeymoon period. Persistence of C-peptide above these levels after 3–5 years from diagnosis is suggestive of Type 2 or monogenic diabetes.

A stimulated blood C-peptide < 0.2 nmol/l (fasting < 0.08 nmol/l and or post-meal urinary C-peptide:creatinine ratio < 0.2 nmol/mmol) confirms absolute insulin deficiency and absolute insulin requirement.

Variations in C-peptide assays, stimulation methods and insulin resistance mean results close to these suggested thresholds should be treated with particular caution.

## Conclusion

In conclusion, C-peptide measurement is an inexpensive, widely available test that may assist the clinical management of diabetes, particularly in insulin-treated patients where there is uncertainty about diabetes subtype.

## Funding sources

AGJ is funded by an NIHR Doctoral Research Fellowship. ATH is employed as a core member of the NIHR Exeter Clinical Research Facility and is an NIHR Senior Investigator and a Wellcome Trust Senior Investigator. ATH receives support from the European Union Grant 22321 (CEED3: Collaborative European Effort to Develop Diabetes Diagnostics). The views given in this paper do not necessarily represent those of NIHR, the NHS or the Department of Health.
